# Modulation of plasma complement by the initial dose of epirubicin/docetaxel therapy in breast cancer and its predictive value

**DOI:** 10.1038/sj.bjc.6606068

**Published:** 2011-01-11

**Authors:** S Garantziotis

**Affiliations:** 1 Laboratory of Respiratory Biology, National Institute of Environmental Health Sciences, PO Box 12233, MD-CU01, 111 TW Alexander Drive, Research Triangle Park, NC 27709, USA; 2 Department of Medicine, University of North Carolina, Chapel Hill, NC, USA

**Sir**,

I read with interest the recent paper by Michlmayr *et al*, 2010, describing the effects of neoadjuvant chemotherapy on serum complement factor expression (Michlmayr *et al*, 2010). The authors also reported increase in levels of the plasma protein inter-*α*-trypsin inhibitor (I*α*I), but they may have failed to appreciate the potential significance of this finding.

I*α*I is not an acute-phase protein, as the authors report in this paper; in fact I*α*I plasma concentration declines during acute inflammation, because of consumption and decreased liver expression (Daveau *et al*, 1993; Opal *et al*, 2007). Moreover, I*α*I heavy chain expression is downregulated in cancers, including breast cancer (Hamm *et al*, 2008). Furthermore, I*α*I does not simply have hyaluronan-binding properties; it inhibits cancer metastasis (Werbowetski-Ogilvie *et al*, 2006; Yagyu *et al*, 2006) and has strong anti-inflammatory properties (Zhuo *et al*, 2004). In fact, we showed that I*α*I inhibits complement activation, both through the classical and the alternative pathways (Garantziotis *et al*, 2007). Thus, the association of increased I*α*I and complement plasma levels is significant for at least two reasons. First, upregulation of I*α*I may be a regulatory mechanism inhibiting complement activation. As baseline I*α*I plasma concentration is substantial (0.1–0.5 mg ml^−1^; Zhuo *et al*, 2004), even a modest relative increase of ∼50%, as the authors report, would mean a significant absolute increase. As the observed increase in plasma I*α*I overrides the expected acute phase decline, powerful induction mechanisms may be assumed. Second, I*α*I is now in production in the United States and will soon be tested in a clinical trial for its effect in sepsis morbidity and mortality. As complement activation appears to have a role in the response to chemotherapy, factors that affect this activation, such as I*α*I, may be interesting as therapeutic agents. Furthermore, I*α*I levels may be predictive of response to treatment as well, as they are in sepsis (Opal *et al*, 2007).

In conclusion, I believe that I*α*I–complement interactions are important in the context of cancer progression and treatment, and these interactions should be highlighted in reference to the recently published paper by Michlmayr *et al*, 2010.
